# *Typhonium giganteum* Lectin Exerts A Pro-Inflammatory Effect on RAW 264.7 via ROS and The NF-κB Signaling Pathway

**DOI:** 10.3390/toxins9090275

**Published:** 2017-09-07

**Authors:** Wei Wang, Hao Wu, Hongli Yu, Xingde Zhang, Guojing Cui, Kuilong Wang, Shanhu Mao, Yaozong Pan

**Affiliations:** 1 School of Pharmacy, Nanjing University of Chinese Medicine, Nanjing 210023, China; zhonglixunta349@163.com (W.W.); xingde2293@126.com (X.Z.); wjwkl@126.com (K.W.); 15295515679@163.com (S.M.); pan_yaozong@126.com (Y.P.); 2 Jiangsu Key Laboratory of Chinese Medicine Processing, Nanjing University of Chinese Medicine, Nanjing 210023, China; 3 Engineering Center of State Ministry of Education for Standardization of Chinese Medicine Processing, Nanjing 210023, China; 4 State Key Laboratory Cultivation Base for TCM Quality and Efficacy, Nanjing University of Chinese Medicine, Nanjing 210023, China; 5 Yancheng Traditional Chinese Medicine Hospital Affiliated to Nanjing University of Chinese Medicine, Yancheng 224000, China; cuiguojing333@126.com

**Keywords:** *Typhonium giganteum* lectin, inflammation, oxidative stress, ROS, NF-κB, apoptosis, necrosis, Bai Fu Zi

## Abstract

*Typhonii rhizoma*, a widely used herb in traditional Chinese medicine, has acute irritating toxicity related to *Typhonium giganteum* lectin (TGL). TGL exhibits acute inflammatory effects, but the underlying molecular mechanisms are largely unknown. This paper is designed to assess the pro-inflammatory response of TGL on RAW 264.7 cells. RAW 264.7 treated with 6.25, 12.5, 25, and 50 µg/mL TGL showed elevated levels of inflammatory factors (TNF-α, IL-1β) and of p-IκB and p-p65, all dose-dependent, indicating that TGL had a substantial inflammatory effect and mobilized the nuclear factor-κB (NF-κB) pathway. All four TGL treatments also induced the up-regulation of reactive oxygen species (ROS) and cytosolic free Ca^2+^ and down-regulation of mitochondrial membrane potential (MMP). The production of cytokines and p-IκB, p-p65 were reduced by N-acetylcysteine (NAC), an ROS scavenger, which somewhat abrogated ROS production. The results showed the TGL-activated inflammatory signaling pathway NF-κB to be associated with the overproduction of ROS. Moreover, 50 μg/mL treatment with TGL led to cell apoptosis after 1 h and increased necrosis over time. These results provided potential molecular mechanisms for the observed inflammatory response to TGL including up-regulation of ROS and cytosolic free Ca^2+^, down-regulation of MMP, the mobilization of the NF-κB pathway, and the subsequent overproduction of pro-inflammatory factors resulting in apoptosis. Long-term stimulation with TGL resulted in strong toxic effects related to inflammation that induced necrosis in macrophages.

## 1. Introduction

*Typhonii rhizoma* is the dry tuber of *Typhonium giganteum* Engl. (Araceae). It is widely used in traditional Chinese medicine to dispel gas and relieve convulsions. However, it is a toxic herb that tastes spicy and can irritate the tongue and throat, leading to soreness, excessive salivation, or even suffocation. It can also cause dermatitis. In a previous study, we found its toxicity to be related to *Typhonium giganteum* lectin (TGL), a type of monocot lectin from the tuber of *T. giganteum*. This lectin demonstrated pro-inflammatory activity, including stimulation of macrophages to generate excessive inflammatory factors (TNF-α, IL-1β), and induction of neutrophil migration [1]. *Pinellia ternata* lectin (PTL), another monocot lectin from the tuber of *Pinellia ternata* (Thunb.) Breit. (Araceae) has similar pro-inflammatory activities involving the overproduction of reactive oxygen species (ROS), mobilization of the NF-κB pathway, and the subsequent release of large quantities of inflammatory factors [2].

Lectins are glycoproteins (sugar-binding proteins) found in plants and animals. They bind to specific sugars and proteins [3,4]. Several studies have shown that certain lectins such as *Helianthus tuberosus* agglutinin [5] or the galactose-binding lectin from *Vatairea macrocarpa* seeds [6] exhibit pro-inflammatory effects, accompanied by neutrophil migration. Acute inflammatory effects have been observed in the paw edema model [5,6,7,8,9,10]. Furthermore, certain lectins may contribute to the overproduction of ROS, leading to autophagy, apoptosis or necrosis [11,12,13,14].

ROS play an important role in inflammation, in which they act as second messengers to mediate the inflammatory response. During inflammation, the production of ROS increases, which contributes to the opening of mitochondrial permeability transition pores, and the release of calcium ions, cytochrome C, and apoptosis-inducing factor. The result in the activation of the NF-κB pathway and caspase3/6/7, which eventually leads to rupture of the mitochondrial membrane and cell death [15,16,17,18]. We reported previously that the pro-inflammatory activity of TGL was related to mobilization of the NF-κB pathway accompanied by overproduction of inflammatory factors [1]; however, the specific mechanisms are still unknown. The current work is designed to establish the mechanism underlying the pro-inflammatory activity of TGL. We hope this work provides new perspectives for the development of the detoxification processing of *Typhonii rhizoma*.

## 2. Results

### 2.1. Extraction, Purification, and Identification of TGL

**Figure 1:**
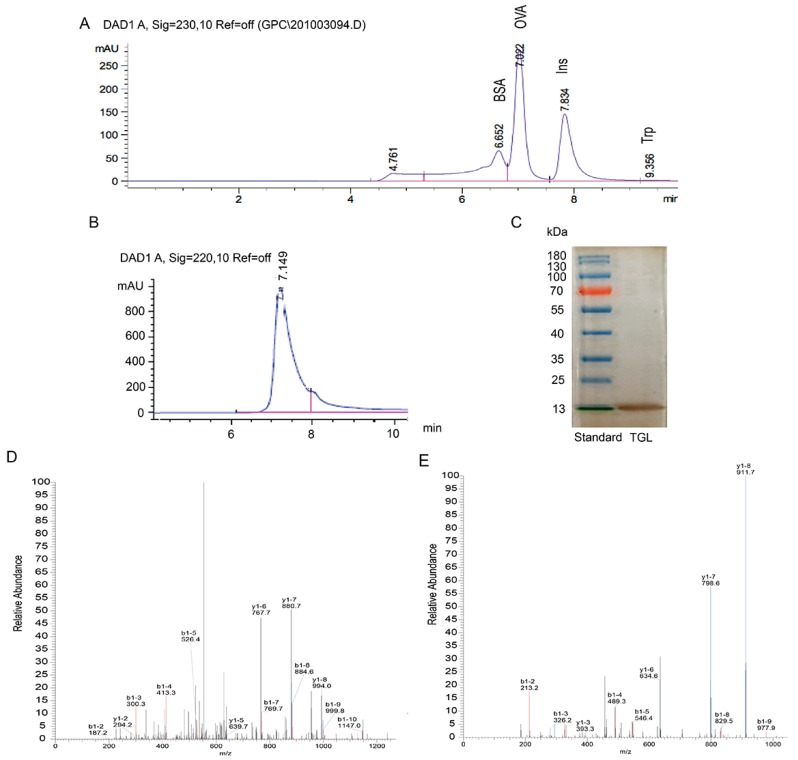
(**A**) Analysis of standard proteins related to bovine serum albumin (BSA, 66.4 kDa), tryptophan (Trp, 0.2 kDa), insulin (Ins, 5.7 kDa) and ovalbumin (OVA,44 kDa) using size-exclusion HPLC; (**B**) *Typhonium giganteum* lectin (TGL) using size-exclusion HPLC; (**C**) Purified TGL (a single band of ≈13 kDa). Silver nitrate was applied to the gel; (**D**) MS/MS analysis of ‘GELIIKDDDFK’ from TGL; (**E**) MS/MS analysis of ‘LVIYGPSVFK’ from TGL.

To purify TGL, we performed the following procedures to extract the crude protein: elution by hydrophobic interactions, ion exchanges, and desalting chromatography. The main chromatogram peak was eluted with NaCl (gradient, 0–1.0 mol/L; flow rate, 2 mL/min). A single peak and band were observed (Figure 1), indicating that the TGL was pure. Then the 13 kDa band was digested by trypsin, accompanied by LC-MS/MS analysis for peptide identification (Figures S1 and S2). We compared database entries, and we observed a single band, identified as TGL, matching those previously reported [1]. The molecular weight of lectins from Araceae are approximately 13 kDa, further supporting this observation [2,19].

### 2.2. Cytokines Released from RAW 264.7 Stimulated by Different Doses of TGL

**Figure 2:**
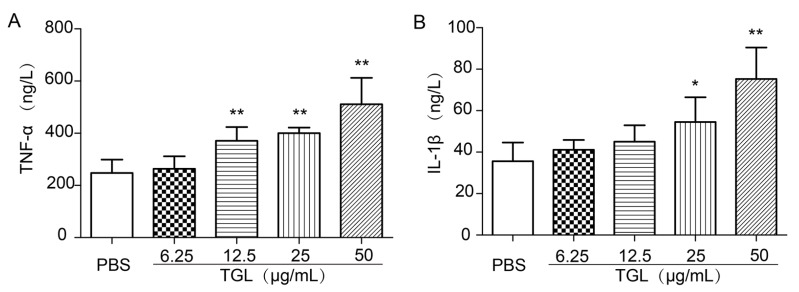
Cytokines released from RAW 264.7 cells treated with *Typhonium giganteum* lectin (TGL) (6.25, 12.5, 25, 50 μg/mL for 1 h). Different doses of TGL induced changes in levels of (**A**) TNF-α and (**B**) IL-1β. Results are mean ± SD (*n* = 6). * *p* < 0.05, ** *p* < 0.01 relative to the group of phosphate-buffered saline (PBS). TNF, tumor necrosis factor; IL, interleukin.

RAW 264.7 cells were treated with 6.25, 12.5, 25, or 50 μg/mL of TGL and then the expressions of inflammatory factors TNF-α and IL-1β were determined. As shown, TGL stimulated macrophages to produce TNF-α and IL-1β in a dose-dependent manner, which suggested that TGL had induced a severe inflammatory response (Figure 2). 

### 2.3. Western Blot Test of p-p65, p65, p-IκB, IκB

**Figure 3:**
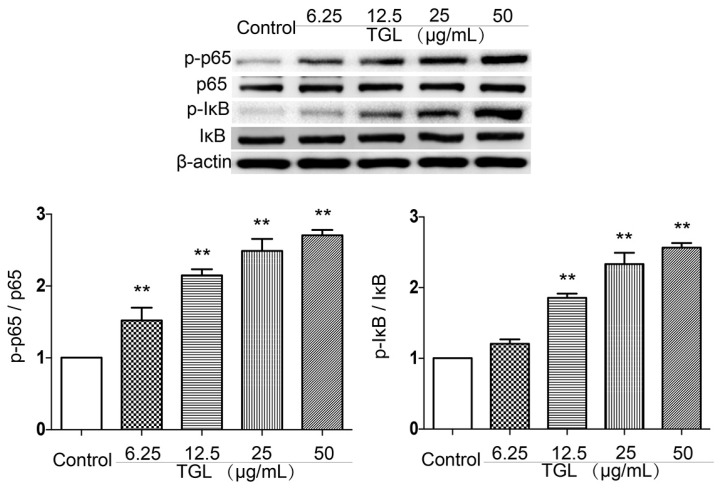
The expression levels of p-p65, p65, p-IκB and IκB in RAW 264.7 cells treated with *Typhonium giganteum* lectin (TGL) (6.25, 12.5, 25, 50 μg/mL for 1 h). Results are mean ± SD (*n* = 3). * *p* < 0.05, ** *p* < 0.01 comparing phosphate-buffered saline (*t*-test).

The NF-κB pathway is involved in inflammatory responses. In the present study, p65 and p-p65, together with IκB, an inhibitory protein of the NF-κB pathway, and p-IκB protein were detected by Western blot test. TGL (6.25, 12.5, 25, 50 μg/mL) was associated with greater expression of p-p65 and p-IκB than in the phosphate-buffered saline (PBS) group (Figure 3), suggesting that the inflammatory response caused by TGL was linked to the NF-κB pathway.

### 2.4. Measurement of ROS, MMP, and the Cytosolic Free Ca^2+^

**Figure 4:**
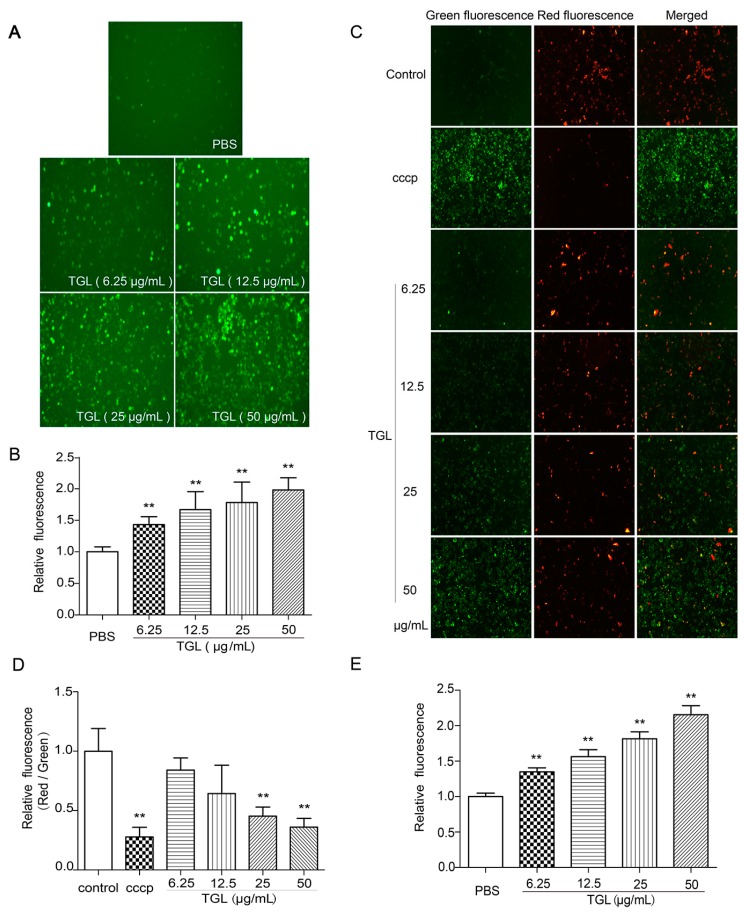
Variation of (**A**,**B**) reactive oxygen species (ROS), (**C**,**D**) mitochondrial membrane potential (MMP), and (**E**) cytosolic free Ca^2+^ induced by RAW 264.7 cells treated with *Typhonium giganteum* lectin (TGL) (6.25, 12.5, 25, 50 μg/mL for 1 h). Results are mean ± SD (*n* = 6). ** *p* < 0.01 comparing phosphate-buffered saline (*t*-test). Images were acquired using a microscope, and the intensity was detected using a fluorescence microplate reader. CCCP, carbonyl cyanide 3-chlorophenylhydrazone.

Being second messengers, ROS are involved in the process of inflammation. After stimulation by an irritant, the level of ROS is increased, followed by mobilization of the NF-κB pathway together with a decrease in mitochondrial membrane potential (MMP) and an increase in cytosolic free Ca^2+^ concentration. We assessed the production of ROS, variation in MMP, and increase in the concentrations of cytosolic free Ca^2+^ using fluorescence microscopy and a fluorescence microplate reader. All doses of TGL (6.25, 12.5, 25, 50 μg/mL) were associated with higher intracellular concentrations of ROS (Figure 4A,B), lower MMP (Figure 4C,D), and greater concentrations of cytosolic free Ca^2+^ than in the control group (Figure 4E). This showed that TGL subjected RAW 264.7 cells to intense oxidative stress, eventually releasing ROS, resulting in the down-regulation of MMP and up-regulation of cytosolic free Ca^2+^.

### 2.5. Cytokines Released after Treatment with NAC (the ROS Scavenger)

**Figure 5:**
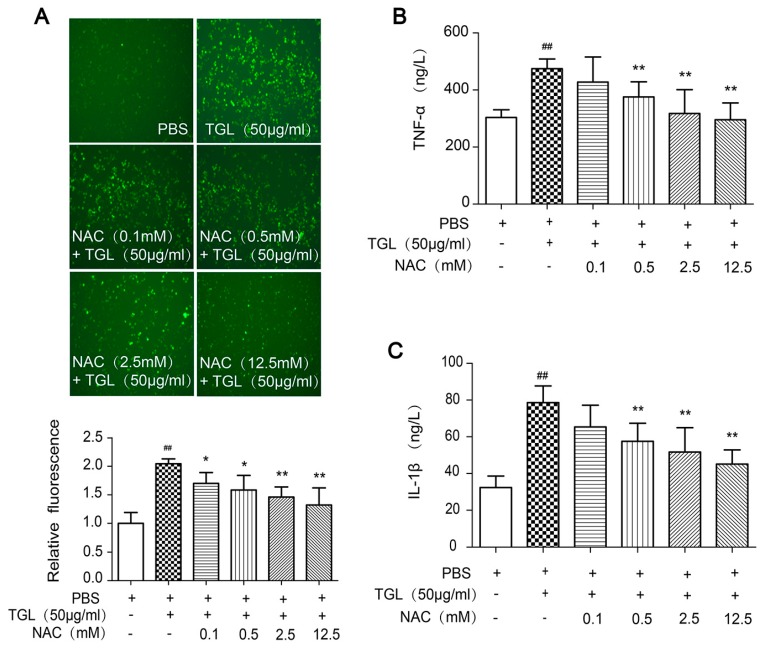
Induction of cytokines released from RAW 264.7 stimulated by *Typhonium giganteum* lectin (TGL) after NAC treatment. (**A**) Variation in ROS induced by TGL with the pretreatment with different doses of NAC; (**B**) Level of TNF-α released from 50 μg/mL TGL-stimulated macrophages after treatment with different doses of NAC; (**C**) Level of IL-1β released from 50 μg/mL TGL-stimulated macrophages after the treatment with different doses of NAC. Results are mean ± SD (n = 6). * *p* < 0.05, ** *p* < 0.01 comparing TGL (50 μg/mL); ^##^ *p* < 0.01 comparing phosphate-buffered saline (*t*-test). TNF, tumor necrosis factor; IL, interleukin; NAC, N-acetylcysteine.

Several reports have demonstrated that ROS is of great importance in inflammation [20,21]. We predicted that ROS generation would trigger the release of cytokines when macrophages were treated with TGL. To test this prediction, we used NAC, the ROS scavenger, to inhibit the generation of ROS (Figure 5A), and then we measured the levels of cytokines released from RAW 264.7 stimulated by TGL, such as TNF-α, IL-1β. Cells treated with NAC showed lower levels of TNF-α and IL-1β expression than those stimulated with 50 μg/mL TGL, in a dose-dependent manner (Figure 5B,C), which indicated that ROS played a crucial part in the inflammatory response caused by TGL.

### 2.6. Western Blot Test of p-p65 and p-IκB after the Treatment with NAC

**Figure 6:**
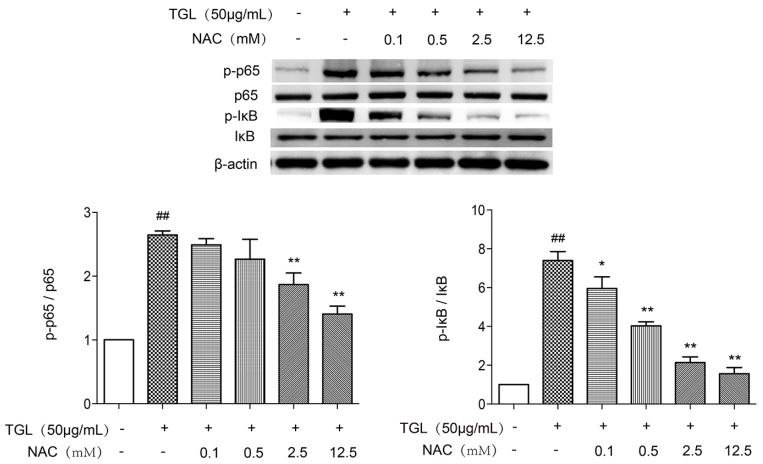
Western blot test of p-p65, p65, p-IκB and IκB of RAW 264.7 stimulated by *Typhonium giganteum* lectin (TGL) after the treatment of NAC. Treatment with all doses of NAC was associated with lower levels of p-p65 and p-IκB expression than in macrophages stimulated with 50 μg/mL TGL alone. Results are mean ± SD (*n* = 3). * *p* < 0.05, ** *p* < 0.01 relative to TGL (50 μg/mL), ^##^ *p* < 0.01 relative to the control group (*t*-test). NAC, N-acetylcysteine.

After treatment with different doses of NAC, the levels of p-p65 and p-IκB expression by RAW 264.7 stimulated by 50 μg/mL TGL were measured by Western blot. As shown in Figure 6, all doses of NAC were associated with less expression of p-p65 and p-IκB. Overall, these results revealed that ROS was of considerable importance to inflammation caused by TGL, involving mobilization of the NF-κB pathway accompanied by overproduction of TNF-α and IL-1β.

### 2.7. Analysis of Death Mode of RAW 264.7 Stimulated by TGL

**Figure 7:**
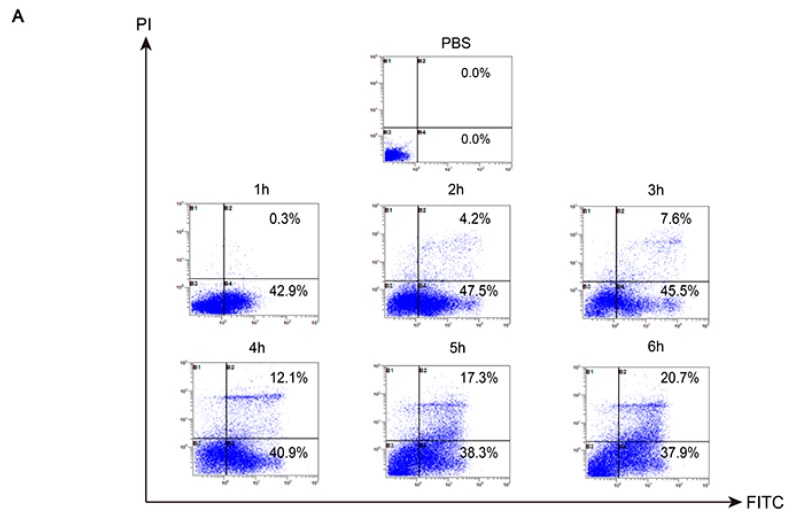

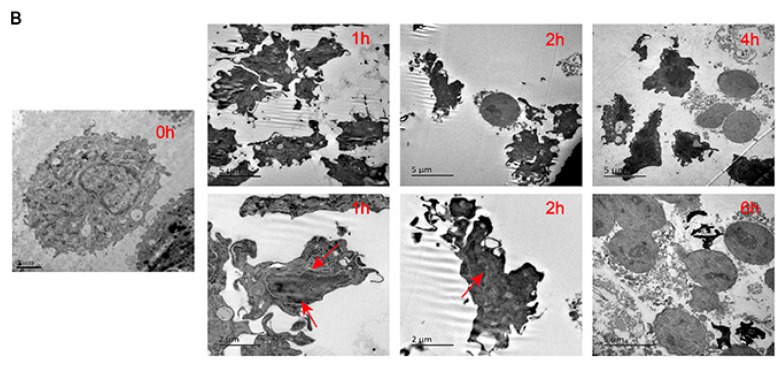
Analysis of the death mode of RAW 264.7 stimulated by TGL via flow cytometry and transmission electron microscope. (**A**) Following the stimulation of 50 μg/mL TGL for 1 h, RAW 264.7 cells underwent apoptosis earlier than in the PBS but did not undergo necrosis earlier. As the treatment period continued (2 to 6 h), the macrophages underwent both necrosis and apoptosis, and the number of necrotic cells increased in a dose-dependent manner; (**B**) After 1 h of stimulation with 50 μg/mL TGL, nuclear chromatin condensation and nuclear shrinkage were observed, which indicated apoptosis. From 2 to 6 h, cellular swelling and cell membrane breakage were visible, indicating necrosis. FITC, Fluorescein isothiocyanate; PI, propidium iodide.

After treatment with 50 μg/mL TGL for 1 h, RAW 264.7 cells underwent early apoptosis rather than necrosis, showing a marked difference from the group treated with PBS (Figure 7A). As TGL treatment continued (2 to 6 h), the macrophages underwent both necrosis and apoptosis, and the number of necrotic cells increased in a dose-dependent manner. As shown in Figure 7B, after stimulation with 50 μg/mL TGL for 1 h, nuclear chromatin condensation and nuclear shrinkage were both evident, which indicated apoptosis. From 2 to 6 h, cellular swelling and cell membrane breakage were seen, indicating necrosis. Collectively, these data revealed that stimulation of 50 μg/mL TGL for 1 h could trigger inflammation and apoptosis in macrophages. From 2 to 6 h, macrophages moved toward necrosis owing to the severe toxicity of TGL.

## 3. Discussion

Lectin is a protein found in plants and animals. It plays an important role in cell recognition and adhesion. It may also have inhibitory activity against the development of Aphidoidea [22,23]. Several studies have indicated that some lectins are toxic to a certain extent, and they can promote the release of inflammatory factors or even cause cell death and inhibit the growth of tumor cells [24,25,26]. *Typhonium giganteum* lectin (TGL) is a protein extracted from *Typhonii rhizoma*; previous studies have demonstrated that TGL exhibited significant pro-inflammatory ability, which could induce the migration of neutrophils and the release of inflammatory factors [1].

This paper was designed to evaluate the mechanism underlying the pro-inflammatory action of TGL. Our results showed that different doses of TGL could induce overproduction of ROS, down-regulation of MMP, and an increase in the concentration of cytosolic free Ca^2+^, accompanied by mobilization of the NF-κB pathway along with the overproduction of inflammatory factors (TNF-α, IL-1β). We treated ROS as an upstream inducer that triggers the mobilization of the NF-κB pathway and subsequently the release of inflammatory factors. Treatment with antioxidants, N-acetylcysteine (NAC), significantly abrogated ROS production, and subsequently inhibited mobilization of the NF-κB pathway and the over-expressions of inflammatory factors. This demonstrated that ROS was of great importance to inflammation induced by TGL. In addition, our data revealed that stimulation of 50 μg/mL TGL for 1 h could trigger the apoptosis in macrophages, and after 2–6 h, macrophage cells became necrotic, indicating that TGL has severe toxicity.

Research has shown that oxidative stress and mitochondrial dysfunction may trigger or even aggravate the occurrence of inflammatory response, with the possibility of forming the inflammasome—e.g., the NLRP1 or NLRP3 inflammasome—leading to local or systemic inflammatory response. Altogether, mitochondrial dysfunction may result in the decrease of MMP, which directly and indirectly led to apoptosis [27]. In this way, mitochondrial dysfunction may disrupt ATP and stimulate the outflow of Ca^2+^ from the mitochondrial matrix, and the release of cytochrome C, which then binds to apoptotic protease-activating factor to initiate caspase cascade and activates caspase-3/7, triggering apoptosis [28]. Necrosis is another pattern of cell death. It plays a key role in inflammation. Under normal circumstances, after infection, the body clears infected cells through apoptosis, but cells may also remain in a state of necrosis if apoptosis is inhibited, which induces the release of cytokines [29]. Our results indicated that after being stimulated by TGL for a long time (2–6 h), the RAW 264.7 macrophages moved toward necrosis rather than apoptosis. We assumed that apoptosis was inhibited through this process, although the mechanism remains to be confirmed.

**Figure 8:**
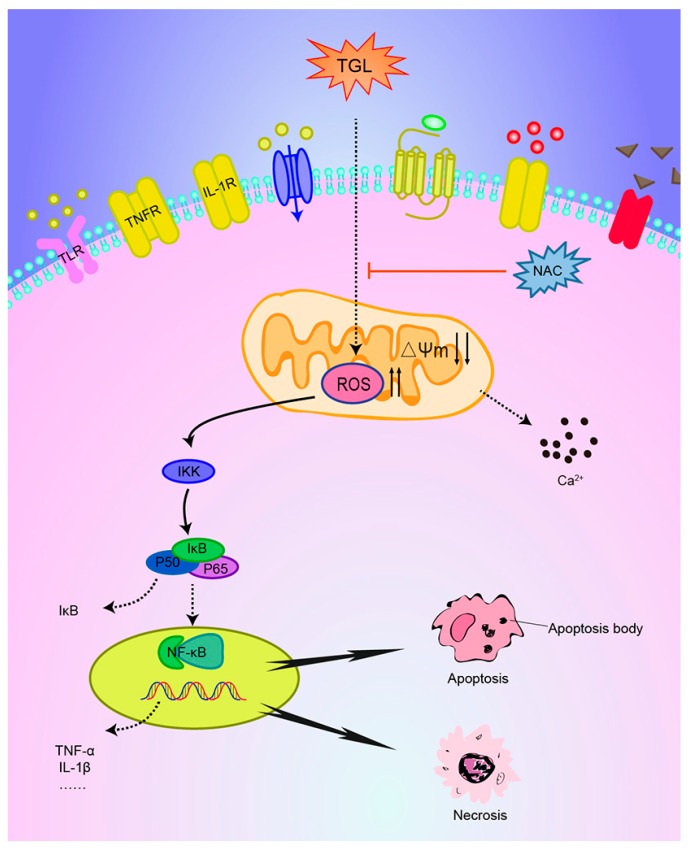
Possible signaling transduction pathways induced by TGL. Different doses of TGL could induce the overproduction of reactive oxygen species (ROS), the down-regulation of mitochondrial membrane potential (MMP), and the up-regulation of cytosolic free Ca^2+^ concentrations, accompanied by activation of the NF-κB pathway and overproduction of inflammatory factors (TNF-α, IL-1β). N-acetylcysteine (NAC), an ROS scavenger, could abrogate ROS production and subsequently inhibit mobilization of the NF-κB pathway and pro-inflammatory response.

Recently, some studies have shown that oxidative stress can be induced in a variety of ways, for example, toll-like receptors [30], tumor necrosis factor receptors [31], and interleukin-1 receptors [32]. Mediated by these receptors, the irritant may trigger oxidative stress along with the NF-κB or MAPKs pathways and the subsequent inflammatory response. Further research is needed to confirm whether oxidative stress caused by TGL is associated with the related receptors (Figure 8).

In summary, these results demonstrate that the pro-inflammatory activity of TGL is linked to oxidative stress that results in the overproduction of ROS and activation of the NF-κB pathway and the over-expressions of TNF-α and IL-1β. These findings are especially noteworthy since they provide a new perspective for the development of the detoxification processing of *Typhonii rhizoma*, which is widely used in traditional Chinese medicine to dispel gas and relieve convulsions.

## 4. Materials and Methods

### 4.1. Plant

The tubers of *T. giganteum* were purchased from Taizhou Gaogang Pieces Factory Co., Ltd., (Taizhou, China) in July 2016 and authenticated by Professor Haobin Hu in Jiangsu Institute for Food and Drug Control.

### 4.2. Reagents and Antibodies

Phenyl Sepharose^TM^ High Performance and Q Sepharose^TM^ High Performance were supplied by GE Healthcare (Uppsala, Sweden). Fetal Bovine Serum (FBS) was from Sciencell (San Diego, CA, USA). Dulbecco’s modified eagle’s medium (DMEM) was from HyClone (Logan, UT, USA). The MMP assay kit with JC-1 and ROS Assay Kit were from Beyotime Institute of Biotechnology (Shanghai, China). Fluo-4 NW Calcium Assay Kits were supplied by Molecular Probes (Eugene, OR, USA). An Annexin V-FITC/propidium Iodide (PI) Apoptosis Detection Kit was from Nanjing KeyGen Biotech. Co., Ltd. (Nanjing, China). Anti-IκB-α and anti-p65 were from Easybio (Beijing, China). Anti-phospho-IκBα and anti-phospho-p65 were from Cell Signaling (Boston, MA, USA). Anti-beta-actin and ELISA kits were supplied by Yifeixue Bio Tech (Nanjing, China).

### 4.3. Extraction of TGL

Tubers of *T. giganteum* (400 g) were cleaned and then crushed with water (800 mL). The mixture was then centrifuged for 30 min at 4 °C. The sediments in the bottom were discarded, while the liquid above was added with ammonium sulfate aqueous solution and centrifuged for 30 min at 4 °C. Then the white solid in the bottom was dissolved with 0.6 mol/L ammonium sulfate aqueous solution and centrifuged for 20 min at 4 °C. The resulting supernatant was added to Phenyl Sepharose^TM^. High Performance column (length, 20 cm; inside diameter, 26 mm). Each column was filled with 75 mL Phenyl Sepharose^TM^ High Performance (GE Healthcare, 17-1082-01). The column was eluted with ammonium sulfate aqueous solution. The mobile phase was composed of A (0.6 mol/L ammonium sulfate aqueous solution) and B (water) with a gradient elution: 0–60 min, 0–100% B. The flow rate was 2 mL/min. The main peak was collected and then added to Q Sepharose^TM^ High Performance column (length, 20 cm; inside diameter, 26 mm). Each column was filled with 75 mL Q Sepharose^TM^ High Performance (GE Healthcare, 17-1014-01). The column was eluted with sodium chloride aqueous solution. The mobile phase was composed of A (water) and B (1.0 mol/L sodium chloride aqueous solution) with a gradient elution: 0–40 min, 0–40% B; 40–50 min, 40–100% B. The flow rate was 2 mL/min. In the end, the main peak was collected, dialyzed and lyophilized. 

### 4.4. SDS-PAGE and SEC-HPLC

The purified TGL was denatured in boiling water for 5 min, and then subjected to SDS-PAGE (15% (*w/v*) acrylamide gel). Silver nitrate was added to the gel. In order to analyze the molecular weight of TGL, standard marker proteins were matched. Then, TGL was checked on an Agilent 1200 system with a Diode Array Detector (DAD) detector by using an Agilent Zorbax GF-450 column (9.4 × 250 mm). The mobile phase was 0.1 mol/L phosphates buffer at a flow rate of 1.5 mL/min.

### 4.5. In-Gel Digestion, LC-Mass Spectromatery (MS)/MS and Database Search [2]

In order to analyze the 13 kDa band shown above further, the band protein was destained, digested and extracted by using certain solvent. Then the extracted peptides were analyzed using LC-ESI-MS-MS. Chromatographic analysis was performed on RP-C_18_ column (0.15 × 150 mm) with temperature at 25 °C. The mobile phase was composed of A containing formic acid-acetonitrile-water (0.1:84:16, *v:v:v*) and B containing formic acid-water (0.1:100, *v:v*) with a gradient elution: 3–15 min, 4–15% A; 15–17 min, 15–50% A; 17–20 min, 50–100% A. The flow rate was 50 nL/min. An electrospray ionization mass spectrometer was used to analyze peptide (mode: positive-ion, voltage: 3.2 KV, temperature: 200 °C). The Arecaceae protein database served as a reference for analysis. 

### 4.6. Cell Culture

RAW 264.7 macrophages were from the Type Culture Collection of the Chinese Academy of Sciences (Shanghai, China). They were cultured in DMEM containing 10% FBS, 100 U/mL penicillin G potassium and 100 μg/mL streptomycin, and then incubated in a CO_2_ incubator at 37 °C. Twelve hours before the formal experiment, the RAW 264.7 cells were cultured in DMEM without FBS to reach the serum starvation state.

### 4.7. Cytokines Stimulated by Different Doses of TGL

RAW 264.7 cells were collected with DMEM, cultivated in 48-wells plates for 2 h. For TNF-α and IL-1β, 5 groups (6 wells/group) were established: group 1, 200 μL/well phosphate buffer solution (PBS) only (control); groups 2–5, 200 μL/well TGL (6.25, 12.5, 25, and 50 μg/mL). After 1 h, the supernatants were collected and then applied to ELISA kits to test TNF-α and IL-1β.

### 4.8. Western Blot Test of p-p65, p65, p-IκB, IκB

To confirm that the overproduction of inflammatory factors was related with the NF-κB pathway, the contents of p-p65, p65, p-IκB, and IκB were assessed by Western blot. RAW 264.7 cells were harvested with DMEM and cultured in 6-wells plates for 2 h. For p-p65, p65, p-IκB, and IκB, 5 groups (3 wells/group) were established: group 1, 500 μL/well PBS only (control); groups 2–5, 500 μL/well TGL (6.25, 12.5, 25, 50μg/mL). After incubation for about 1 h, the supernatants were discarded, and then the cells were extracted using Radio-ImmunoprecipitationAssay (RIPA) lysis buffer, the concentrations of the extracted proteins were measured using the bicinchoninic acid (BCA) assay. The protein solutions were denatured in boiling water for 5 min, mixed with 5X loading buffer, and applied to a 10% (*w/v*) acrylamide gel. They were then transferred onto a polyvinylidene difluoride (PVDF) membrane. After blocking of 5% Albumin from bovine serum (BSA) for 2 h, the membrane was applied to anti-mouse p-p65, p65, p-IκB, IκB and β-actin, respectively, after bonding with horse radish peroxidase (HRP)-conjugated goat anti-mouse IgG antibody for approximately 2 h. The protein was then visualized with a LAS-4000 mini Luminoimage analyzer (FujiFilm, Tokyo, Japan) and ECL chemiluminescence detection kit.

### 4.9. Measurement of ROS

An oxidation-sensitive fluorescent probe DCFH-DA was used to analyze the expression of ROS. RAW 264.7 cells were collected with DMEM, cultivated in 48-wells plates for 2 h. For ROS, 5 groups (6 wells/group) were established: group 1, 200 μL/well PBS only (control); groups 2–5, 200 μL/well TGL (6.25, 12.5, 25, or 50 μg/mL). After treatment for 1 h, DCFH-DA was added to cells for 20 min and the intensity was measured with an excitation wavelength of 488 nm and an emission wavelength of 525 nm.

### 4.10. Detection of Mitochondrial Membrane Potential (MMP) Variation

A 5,5′,6,6′-Tetrachloro-1,1′,3,3′-tetraethyl-imidacarbocyanine (JC-1) probe was used for MMP. RAW 264.7 cells were collected with DMEM, cultivated in 48-wells plates for 2 h. After treatment with TGL (0, 6.25, 12.5, 25, and 50 μg/mL with 6 wells/group) for 1 h or carbonyl cyanide 3-chlorophenylhydrazone (CCCP) for 20 min (a positive control), JC-1 staining solution was added to each well for 20 min. After three rounds of washing, RAW 264.7 cells were analyzed by using a fluorescence microscope (Leica, Wetzlar, Germany), and then using a fluorescence microplate reader (PerkinElmer, Waltham, MA, USA) to determine the red–green ratios.

### 4.11. Measurement of Cytosolic Free Ca^2+^ Concentrations

RAW 264.7 cells were collected with DMEM and cultivated in black 96-wells plates for 2 h. To test the levels of cytosolic free Ca^2+^, 5 groups (6 wells/group) were established: group 1, 200 μL/well PBS only (control); groups 2–5, 200 μL/well TGL (6.25, 12.5, 25, or 50 μg/mL). After incubation for about 1 h, RAW 264.7 cells were loaded with Fluo-4. After 30 min, the cells were analyzed using fluorescence microplate reader (PerkinElmer, Waltham, MA, USA) with an excitation at 494 nm and emission at 516 nm.

### 4.12. Cytokines Released after Treatment with NAC (the ROS Scavenger)

To study the involvement of particular ROS in inflammation caused by TGL, the ROS scavenger N-acetylcysteine (NAC) was used. RAW 264.7 cells were collected with DMEM, cultivated in 48-wells plates for 2 h. After treatment with NAC (0, 0.1, 0.5, 2.5, 12.5 mM with 6 wells/group) for 20 min, TGL (50 μg/mL) was added to each well. After incubation about 1 h, the supernatants were collected and then applied to ELISA kits to test TNF-α and IL-1β.

### 4.13. Western Blot Test of p-p65 and p-IκB after Treatment with NAC

RAW 264.7 cells were collected with DMEM and cultivated in 6-wells plates for 2 h. After treatment with NAC (0, 0.1, 0.5, 2.5, 12.5 mM with 3 wells/group) for 20 min, TGL (50 μg/mL) was added to each well. After incubation about 1 h, the supernatants were discarded, and the cells were washed with PBS. The extraction of total proteins from RAW 264.7 and Western blot test of p-p65, p65, p-IκB, IκB and β-actin were treated as described in Section 4.8.

### 4.14. Analysis of the Death Mode of RAW 264.7 Stimulated by TGL via Flow Cytometry and Transmission Electron Microscopy

In order to analyze the mode of death RAW 264.7 cells stimulated with TGL, the FITC and PI probes was used. That is, RAW 264.7 cells were treated with TGL (50 μg/mL) for 1, 2, 3, 4, 5, and 6 h. RAW 264.7 cells were then washed with PBS and then collected with a specific buffer. Then, FITC and PI probes were added to each sample in accordance with the kit instructions. After incubation about 20 min, the RAW 264.7 cells were test by flow cytometer (Beckman, Brea, CA, USA).

RAW 264.7 cells were collected with DMEM, cultivated in 6-wells plates for 2 h. TGL (50 μg/mL) was added to each well. After treatment for 1, 2, 3, 4, 5, and 6 h, the cells were fixed with 4% glutaraldehyde precooled at 4 °C for 10 h. Then, cells were washed three times with PBS, fixed with 1% osmic acid for 1 h. The cells were washed three times with PBS, dehydrated with acetone, and then Epon812 embedding medium was used for embedding, accompanied by polymerization under the condition of 35 °C, 12 h; 45 °C, 12 h; 60 °C,12 h. After that, the embedded blocks were cut into slices for about 50 nm. Finally, cells were tested using a transmission electron microscope.

### 4.15. Statistical Analysis

SPSS v. 16.0 for Windows (SPSS, Chicago, IL, USA) was used for *t*-test. Numerical data were expressed as mean ± SD. Results with *p* < 0.05 were considered statistically significant.

## Supplementary Materials

The following are available online at www.mdpi.com/2072-6651/9/9/275/s1, Figure S1: MS/MS analysis of ‘GELIIKDDDFK’ from TGL, Figure S2: MS/MS analysis of ‘LVIYGPSVFK’ from TGL.
