# Supplementary material for: Typhonium giganteum Lectin Exerts A Pro-Inflammatory Effect on RAW 264.7 via ROS and The NF-κB Signaling Pathway
Source: Toxins (Basel). 2017 Sep 7;9(9):275. doi: 10.3390/toxins9090275 (PMC5618208; doi:10.3390/toxins9090275)
Supplement: Supplementary file 1 [file toxins-09-00275-s001.pdf]

Supplementary Materials: *Typhonium giganteum* Lectin Exerts A Pro-Inflammatory Effect on RAW 264.7 via ROS and The NF-κB Signaling Pathway

Wei Wang, Hao Wu, Hongli Yu, Xingde Zhang, Guojing Cui, Kuilong Wang, Shanhu Mao and Yaozong Pan

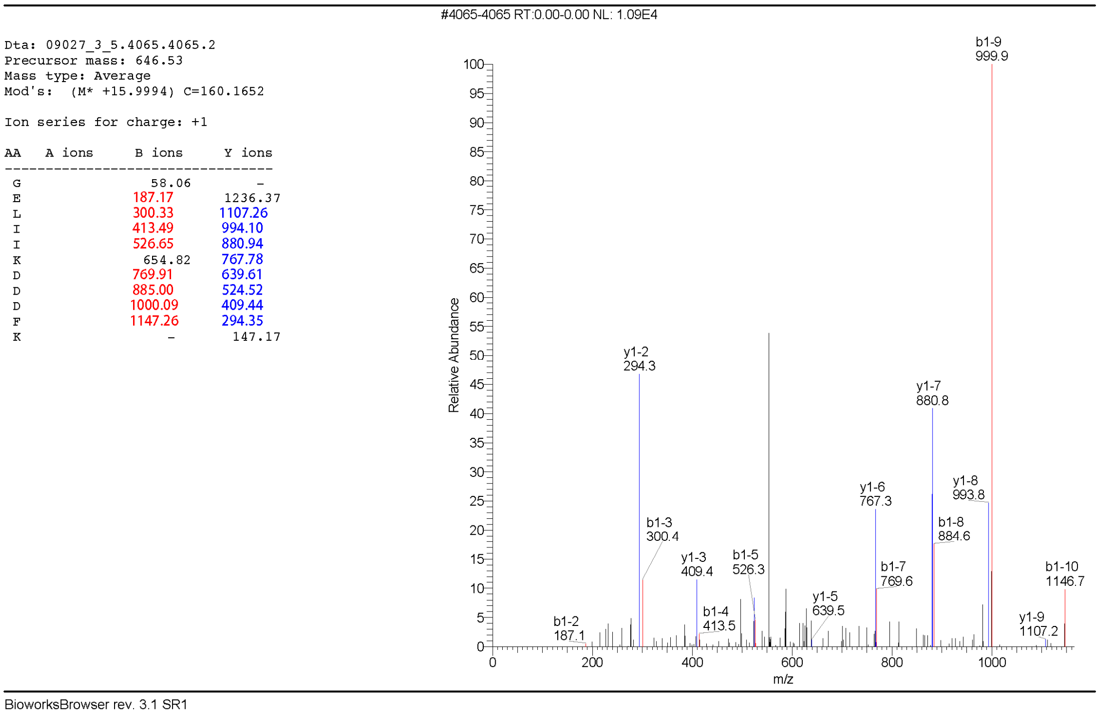

Dta: 09027_3_5.4065.4065.2
Precursor mass: 646.53
Mass type: Average
Mod's: (M* +15.9994) C=160.1652

Ion series for charge: +1

AA	A ions	B ions	Y ions
G		58.06	-
E		187.17	1236.37
L		300.33	1107.26
I		413.49	994.10
I		526.65	880.94
K		654.82	767.78
D		769.91	639.61
D		885.00	524.52
D		1000.09	409.44
F		1147.26	294.35
K		-	147.17

Figure S1. MS/MS analysis of 'GELIIKDDDFK' from TGL.

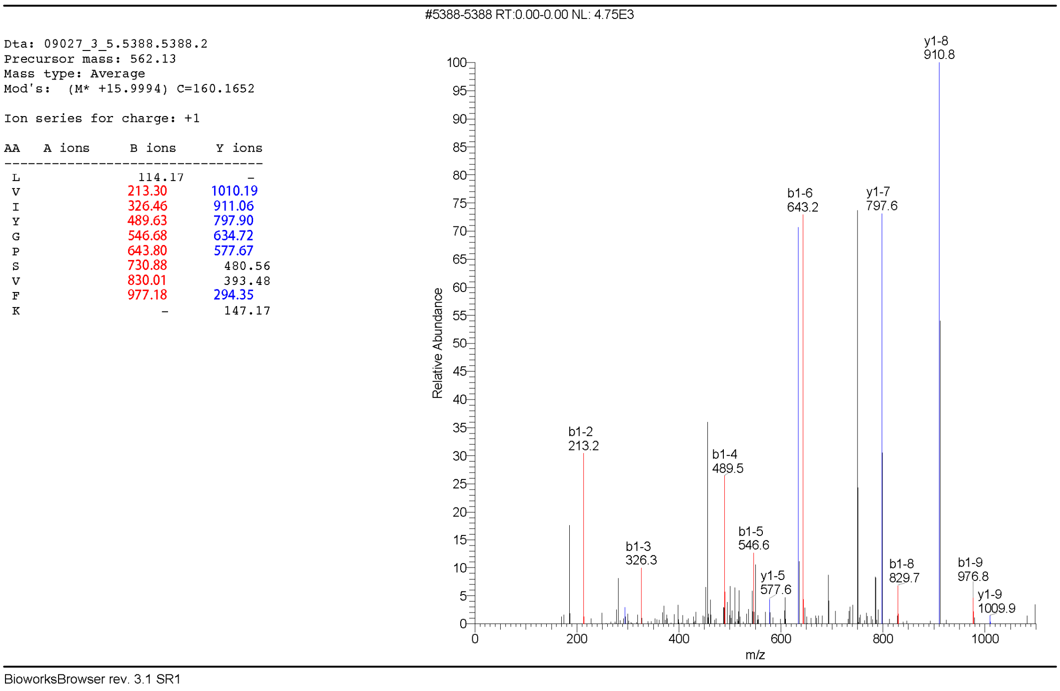

Dta: 09027_3_5.5388.5388.2
Precursor mass: 562.13
Mass type: Average
Mod's: (M* +15.9994) C=160.1652

Ion series for charge: +1

AA	A ions	B ions	Y ions
L		114.17	-
V		213.30	1010.19
I		326.46	911.06
Y		489.63	797.90
G		546.68	634.72
P		643.80	577.67
S		730.88	480.56
V		830.01	393.48
F		977.18	294.35
K		-	147.17

Figure S2. MS/MS analysis of 'LVIYGPSVFK' from TGL.
